# Functional α7β2 nicotinic acetylcholine receptors expressed in hippocampal interneurons exhibit high sensitivity to pathological level of amyloid β peptides

**DOI:** 10.1186/1471-2202-13-155

**Published:** 2012-12-29

**Authors:** Qiang Liu, Yao Huang, Jianxin Shen, Scott Steffensen, Jie Wu

**Affiliations:** 1 Divisions of Neurology, Barrow Neurological Institute, St. Joseph's Hospital and Medical Center, Phoenix, Arizona, 85013-4496, USA; 2 Department of Obstetrics and Gynecology, St. Joseph's Hospital and Medical Center, Phoenix, Arizona, 85004, USA; 3 Department of Physiology, Shantou University Medical College, Guangdong, 515041, China; 4 Department of Psychology, Brigham Young University Provo, Provo, UT, 84602, USA; 5 Departments of Bioengineering, Arizona State University, Tempe, AZ, 85287, USA

**Keywords:** Nicotinic acetylcholine receptor, Amyloid, Hippocampal interneuron, Patch-clamp, Acutely dissociated neuron

## Abstract

**Background:**

β-amyloid (Aβ) accumulation is described as a hallmark of Alzheimer’s disease (AD). Aβ perturbs a number of synaptic components including nicotinic acetylcholine receptors containing α7 subunits (α7-nAChRs), which are abundantly expressed in the hippocampus and found on GABAergic interneurons. We have previously demonstrated the existence of a novel, heteromeric α7β2-nAChR in basal forebrain cholinergic neurons that exhibits high sensitivity to acute Aβ exposure. To extend our previous work, we evaluated the expression and pharmacology of α7β2-nAChRs in hippocampal interneurons and their sensitivity to Aβ.

**Results:**

GABAergic interneurons in the CA1 subregion of the hippocampus expressed functional α7β2-nAChRs, which were characterized by relatively slow whole-cell current kinetics, pharmacological sensitivity to dihydro-β-erythroidine (DHβE), a nAChR β2* subunit selective blocker, and α7 and β2 subunit interaction using immunoprecipitation assay. In addition, α7β2-nAChRs were sensitive to 1 nM oligomeric Aβ. Similar effects were observed in identified hippocampal interneurons prepared from GFP-GAD mice.

**Conclusion:**

These findings suggest that Aβ modulation of cholinergic signaling in hippocampal GABAergic interneurons via α7β2-nAChRs could be an early and critical event in Aβ-induced functional abnormalities of hippocampal function, which may be relevant to learning and memory deficits in AD.

## Background

Aβ accumulation is considered to be a hallmark of Alzheimer’s disease (AD) and responsible for synaptic deficits and neuronal degeneration in AD [1]. Although AD is considered to be a result of aberrant Aβ production [2], the underlying mechanisms of how Aβ deposition contributes to neuronal damage remain unclear. Nicotinic acetylcholine receptors containing α7 subunits (α7-nAChRs) regulate development, differentiation, cognition and pathophysiology of the central nervous system [3-7]. In hippocampus, the highest levels of α7-nAChRs are most commonly found on GABAergic interneurons [8-10], suggesting their potential role in modulating the physiology of the hippocampus, an area of the brain implicated in learning/memory.

Accumulating lines of evidence indicate that α7-nAChRs are involved in AD pathology, and suggest possible pathophysiological links between Aβ and nAChRs. Wang et al. [11,12] first reported high affinity binding of Aβ_1-40_ and Aβ_1-42_ to α7-nAChRs. Two other groups subsequently reported direct and functionally-relevant interactions of Aβ_1-42_ with α7-nAChRs [9,13]. Thereafter, several groups, including ours, reported the effects of Aβ on α7-nAChRs [14-17]. Although some reports demonstrate an activating effect of Aβ on heterologously transfected and native nAChRs [18,19], most *in vitro* studies show an inhibitory effect of acute application of Aβ to neural model preparations including native α7-nAChR expressing cells in culture, in brain slices, or transfected cell-line with α7-nAChRs [9,13,17,20].

Recent findings have demonstrated that neuronal circuits exhibit hyper-excitation rather than hypo-excitation in both AD patients and model animals [21-26]. Given that α7-nAChRs expressed on hippocampal interneurons are inhibited by Aβ, a disruption of these cholinergic inputs to hippocampal interneurons may not only affect neurotrophic support to these interneurons and cause neuronal degeneration, but may also cause disinhibition of pyramidal neurons in hippocampus and lead to neuronal network hyper-excitation due to a disrupted homeostatic regulation [23,24,27].

In our previous studies, we discovered a novel type of heteromeric α7β2-nAChR in rodent basal forebrain cholinergic neurons that is sensitive to Aβ, implying that α7β2-nAChRs might be a critical target for AD pathogenesis [17]. In the present study, we extend our previous work to investigate whether or not this heteromeric α7β2-nAChR is also expressed in hippocampal GABAergic interneurons and to determine its sensitivity to pathologically relevant concentration of Aβ oligomer by utilizing electrophysiological, histological and genetic engineering approaches. Our findings suggest the existence of functionally heteromeric α7β2-nAChRs in hippocampal GABAergic interneurons and these α7β2-nAChRs are sensitive to low nanomolar concentrations of Aβ oligomer.

## Results

### Functional α7-containing nAChRs in hippocampal CA1 interneurons

**Figure 1:**
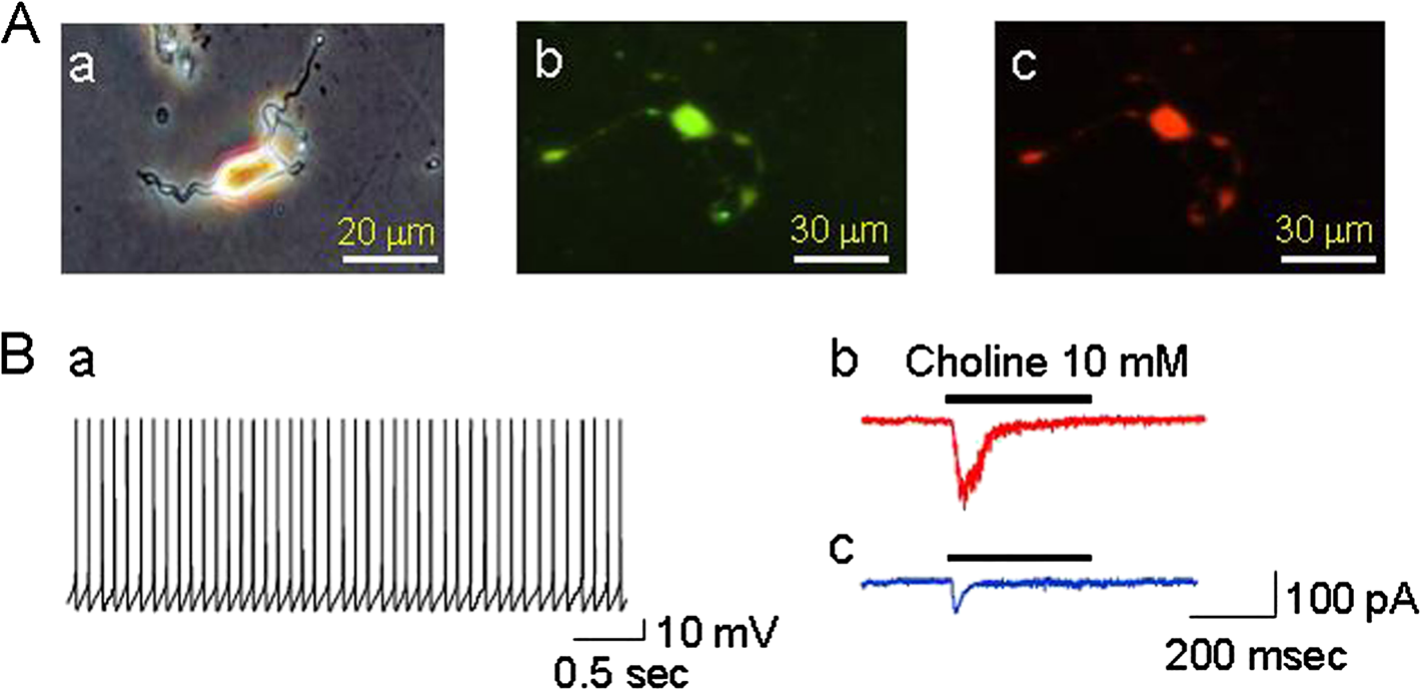
Functional α7*-nAChRs in acutely dissociated mouse hippocampal interneurons. Aa: Phase contrast microscopic image of acutely dissociated typical interneuron from the rat hippocampal CA1 area. **A**b: Biocytin was injected into a recorded interneuron and stained with streptoavidin. **A**c: Double-staining of biocytin-injected neuron with GAD 67 antibody. Scale bar for panel Aa = 20 μm, for Ab and Ac = 30 μm. **B**a: Unlike pyramidal cells, hippocampal interneurons exhibit relatively high frequency spontaneous action potentials. **B**b: α7-nAChR agonist-induced whole-cell currents in an acutely dissociated interneuron (red trace) and a non-interneuron (blue trace), indicating functional α7-nAChRs expressed in these neurons.

To identify hippocampal GABAergic interneurons, tissue was punched from CA1 and the cells were acutely dissociated and selected based on their morphology. As shown in Figure 1, unlike pyramidal neurons, the typical hippocampal interneurons display bipolar or multipolar configurations (Figure 1Aa) and relatively rapid spontaneous action potential firings (Figure 1Ba). Figure 1Bb shows that 10 mM choline (a selective α7-nAChR agonist) induced a typical inward current from a hippocampal interneuron (red trace) and non-interneuron (blue trace) acutely dissociated from CA1 area. To confirm that the recorded neuron was GABAergic, biocytin was microinjected through the pipette solution and followed by immunostaining with streptoavidin (Figure 1Ab, green) and GAD 67 antibody (Figure 1Ac, red, a marker for GABAergic neurons).

### Nicotinic receptor α7 and β2 subunits are co-expressed and co-assembled in mouse hippocampus

**Figure 2:**
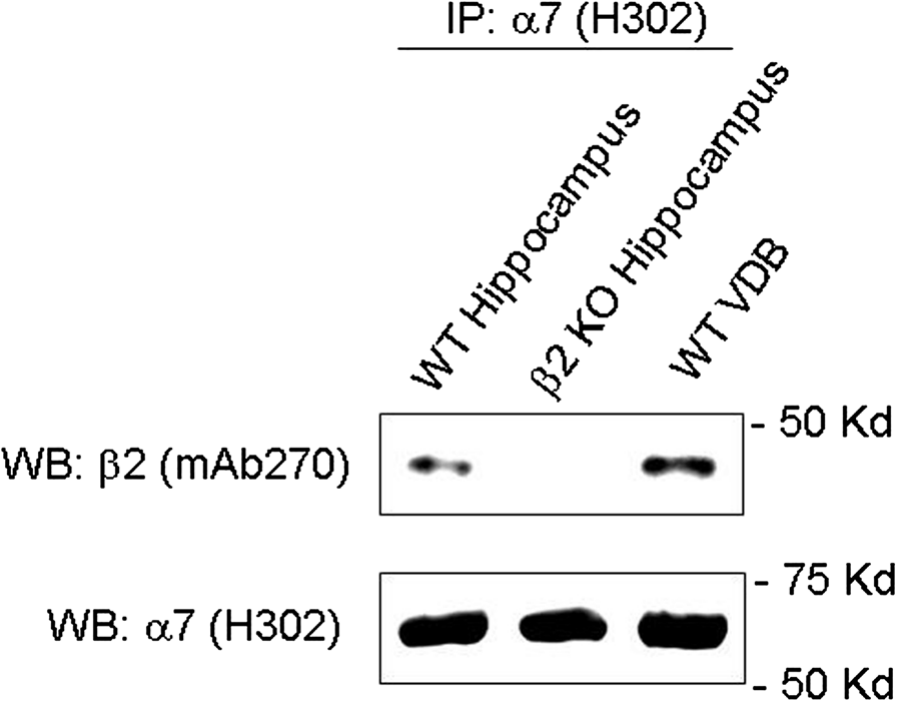
**Co-assembly of α7β2-nAChRs in the hippocampal CA1.** Protein extracted from wild type mouse hippocampus CA1 (lane 1) or β2 KO mouse hippocampus CA1 (lane 2) or from VDB of wild type mice (lane 3) were immunoprecipitated (IP) with rabbit anti-α7 antiserum H302 (lanes 1, 2, and 3). The eluted proteins from the precipitates were analyzed by immunoblotting (IB) with rat monoclonal anti-β2 subunit antibody mAb270 (top) or rabbit anti-α7 antiserum H302 (bottom).

To test the possibility that nAChR α7 and β2 subunits are co-expressed and co-assembled in hippocampus, we performed co-immunoprecipitation (co-IP) assays using nAChR α7 and β2 subunit-specific antibodies. The specificity of these antibodies has been described previously [17]. Protein extracts from wild type or β2 knockout mice hippocampus or vertical diagonal band (VDB) tissues (collected from mice aged between 18 and 22 postnatal days identical to electrophysiology recordings) were subjected to immunoprecipitation (IP) (Figure 2) with a rabbit anti-nAChR α7 subunit antibody (H302) followed by immunoblotting (IB) with a rat anti-nAChR β2 subunit monoclonal antibody (mAb270). As indicated in Figure 2, the β2 subunit was readily detected immunologically in anti-α7 immunoprecipitates from either hippocampus or VDB in wild type mice but not from hippocampus in β2 knockout mice under the same experimental conditions (Figure 2, lanes 1 2, and 3). Reprobing the same blot with the rabbit anti-α7 antibody (H302) verified that similar amounts of α7 subunits were precipitated from both hippocampus and VDB tissues of wild type mice (Figure 2, lanes 1 and 3). Collectively, considering the fact that both α7 and β2 subunits are mostly expressed on hippocampal interneurons [10,28,29], these results suggest that nAChR α7 and β2 subunits are very likely co-assembled in mouse hippocampal interneurons.

### Determination of Aβ forms

**Figure 3:**
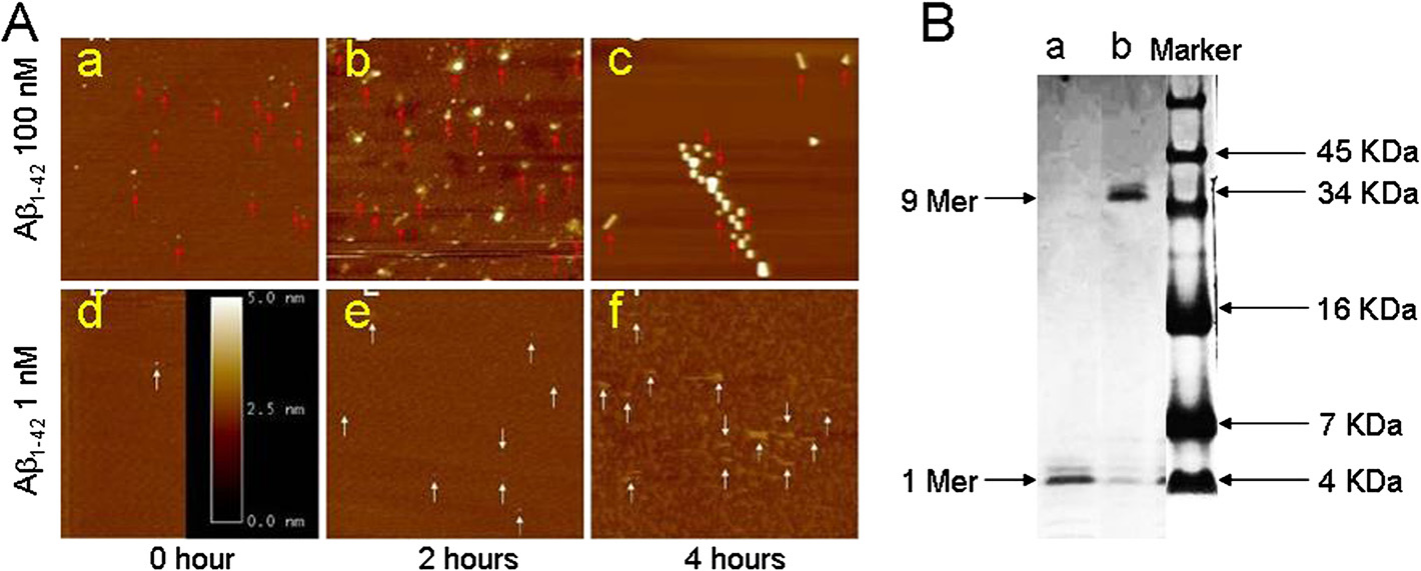
**AFM image shows the forms of Aβ**_**1-42 **_**after preparation. Aa-c**: 100 nM Aβ after preparation at 0, 2 and 4 hrs. Red arrows indicate the small oligomers (**A**a), small and large oligomers (**A**b) and large oligomer and protofibriles (**A**c). **A**d-f: 1 nM Aβ at 0, 2 and 4 hrs after preparation. **B**: When dissolved with DMSO, monomer is the major form of Aβ after 2 hrs aggregation (Lane a). 9 Mer is the major form of Aβ, when dissolved with ACSF, after 2 hrs aggregation (Lane b).

In this study, AFM was used to monitor aggregation forms of Aβ. Aβ was dissolved in distilled water to a concentration of 100 μM and then diluted to desired concentrations. Preparations of Aβ at 100 nM (0–4 hr) contained small oligomers (Figure 3A), small and large oligomers (Figure 3B) and large oligomer and protofibriles (Figure 3C) in AFM images. 1 nM Aβ at 0, 2 and 4 hr after preparation mostly form smaller oligomers. In all experiments within this study, Aβ was used within 4 hr before discarded.

Although AFM is able to distinguish oligomer from fibril or monomer (since monomer is not detectable) based on their sizes, it can not specify the exact forms of oligomer during the aggregation state. To examine what forms of Aβ peptides were present in our samples, we utilized electrophoresis to test the exact forms of oligomer during aggregation state. As shown in Figure 3G, after 2 hrs aggregation of Aβ, if dissolved with water, the major form is 9 mer, while the major form of DMSO dissolved Aβ after 2 hrs aggregation remains in the monomer state.

### Pharmacological profiles of functional α7*-nAChRs in hippocampal CA1 interneurons

**Figure 4:**
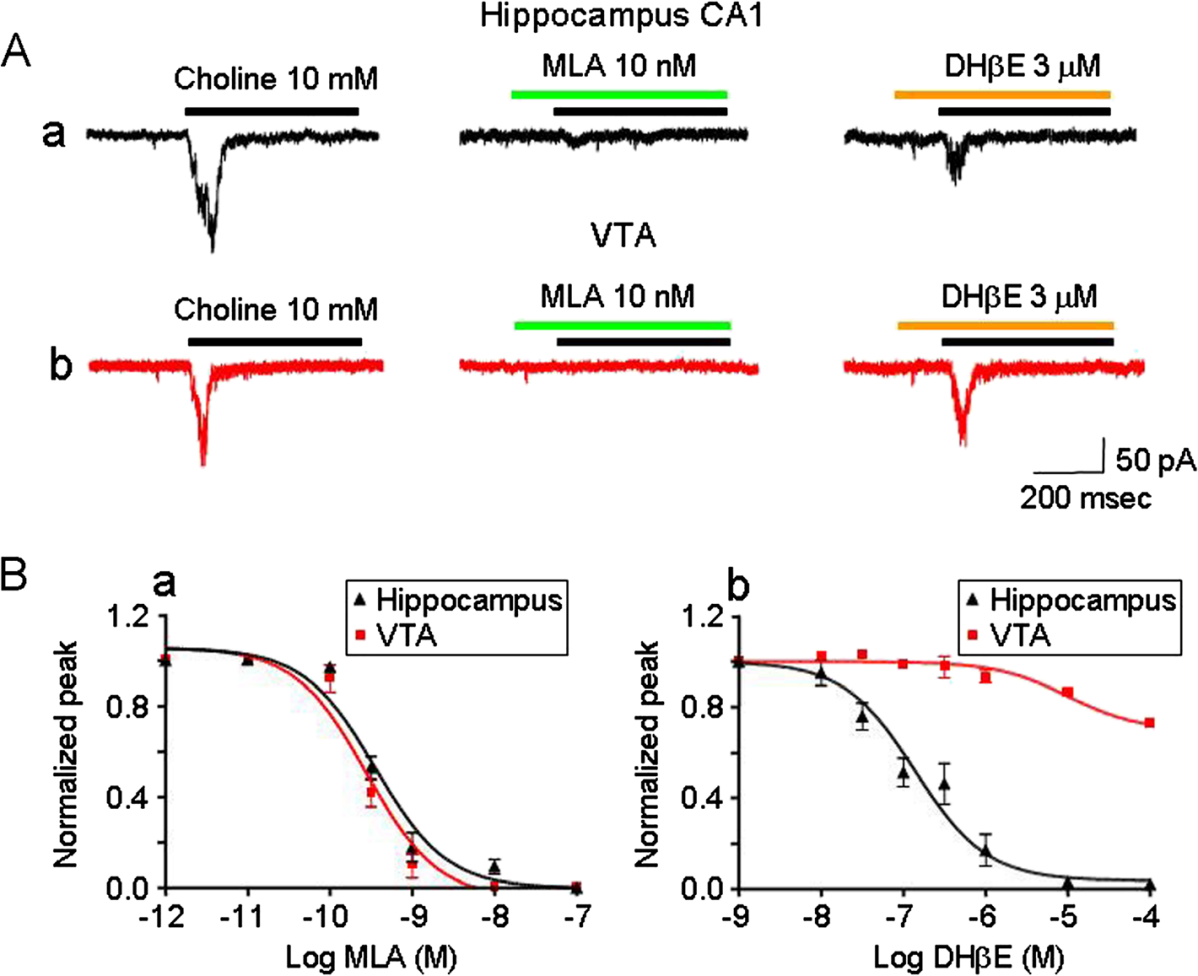
**Pharmacological properties of α7β2-nAChRs expressed in hippocampal CA 1 interneurons. A**. A typical trace of inhibitory effects of selective α7-nAChR antagonist (MLA), or β2-nAChR antagonist (DHβE) on choline-induced whole cell currents recorded from a hippocampal CA1 interneuron (a) and a VTA DAergic neuron (b) at a holding potential of −60 mV. **B**. Analysis of concentration-inhibition curves of MLA shows that after pre-incubation for 2 min, 10 mM choline-induced whole-cell currents in hippocampal CA1 interneurons and VTA DA neurons were not significantly different (**B**a). However, DHβE significantly inhibited choline-induced whole-cell currents from hippocampal CA1 interneurons but not those from VTA DA neurons (Bb, **p < 0.01, *t-test*).

Pharmacological approaches were used to characterize and compare features of functional nAChRs expressed in hippocampal CA1 interneurons and in ventral tegmental area (VTA) dopamine (DA) neurons since VTA DA neurons are known to express homomeric α7-nAChRs [30,31]. The α7-nAChR-selective antagonist methyllycaconitine (MLA) showed similar antagonist potency toward choline-induced currents in either hippocampal CA1 (Figure 4Aa) interneurons or VTA DA neurons (Figure 4Ab). Analysis of concentration-inhibition curves by preincubation with MLA for 2 min (Figure 4Ba) yielded IC_50_ values and Hill coefficients of 0.5 nM and 1.2 for hippocampal interneurons (n = 6) and 0.3 nM and 1.0 for VTA DA neurons (n = 6, hippocampus vs. VTA *p* > 0.05), respectively. However, the β2*-nAChR-selective antagonist DHβE was ~500-fold more potent as an inhibitor for choline-induced current in hippocampal interneurons (Figure 4Ba) than that in VTA DA neurons (Figure 4Bb). IC_50_ values and Hill coefficients for DHβE-induced inhibition were 0.18 μM and 0.8, for hippocampal interneurons (n = 6), and >100 μM and 0.5 for VTA neurons (n = 6; hippocampus vs. VTA, p < 0.001; Figure 4Bc), respectively. These results are consistent with the hypothesis that functional α7*-nAChRs on hippocampal interneurons likely contain DHβE–sensitive β2 subunits.

### Aβ inhibits α7β2-nAChRs expressed on acutely dissociated hippocampal interneurons

**Figure 5:**
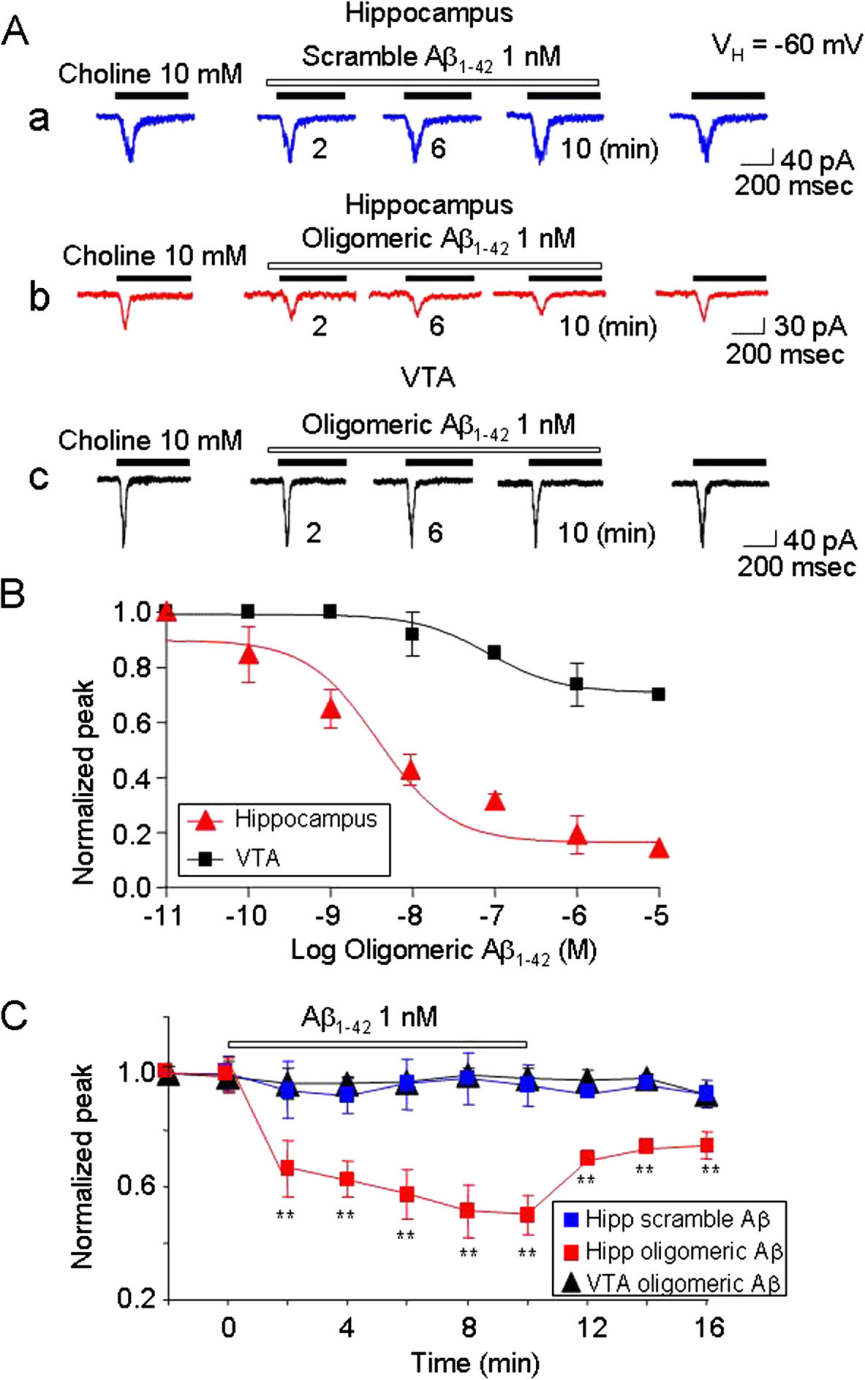
**Effects of 1 nM Aβ on α7β2-nAChRs expressed in acutely dissociated hippocampal interneurons or VTA dopamine neurons.** Typical traces illustrate the effects of 1 nM scrambled Aβ (**A**a) or oligomeric Aβ Ab) on 10 mM choline-induced currents recorded from hippocampal interneurons and from VTA DA neurons (**A**c). The membrane potential was held at −60 mV for these recordings. **B:** Concentration-response profile shows that choline-induced currents in hippocampus CA1 interneurons were more sensitive to block by Aβ (at the indicated concentrations in M after pre-exposure for 2 min) than those in VTA neurons. **C:** Summarized results of experiments as shown in **A**. Each symbol was averaged from 6 cells tested. The vertical bars indicate Mean ± SE. **p < 0.01, *t-test.*

To test the sensitivity to Aβ of α7-nAChRs in hippocampal interneurons, we examined the effects of 1 nM Aβ_1-42_ (with predominantly oligomers) on these receptors. The experimental protocol involved repeated, acute challenges with 10 mM choline spaced at a minimum of 2-min intervals. During a continuous exposure to 1 nM Aβ_1-42_ starting just after an initial choline challenge and continuing for 10 min, responses to choline challenges were progressively inhibited with time by 1 nM Aβ_1-42_ in hippocampal interneurons, although reversibly as demonstrated by response recovery after 6 min of peptide washout (Figure 5Ac). By contrast, exposure to 1 nM scrambled Aβ (as a control peptide) had no effect (Figure 5Ab). Choline-induced currents in dissociated VTA DA neurons were not sensitive to 1 nM Aβ oligomeric treatment (Figure 5Aa). Concentration-response profile shows that choline-induced currents in hippocampus CA1 interneurons were more sensitive to block by Aβ (at the indicated concentrations in M after pre-exposure for 2 min) compared to those in VTA neurons (Figure 5B). Quantitation of three replicate experiments of 6 cells (Figure 5C) confirmed that Aβ, even at 1 nM concentration, specifically inhibits putative α7β2-nAChR function on hippocampal GABAergic interneurons, but not the function of homomeric α7-nAChRs on VTA DA neurons.

### Aβ inhibits choline-induced responses on acutely dissociated hippocampal GAD-positive interneurons prepared from GFP-GAD knock-in mice

**Figure 6:**
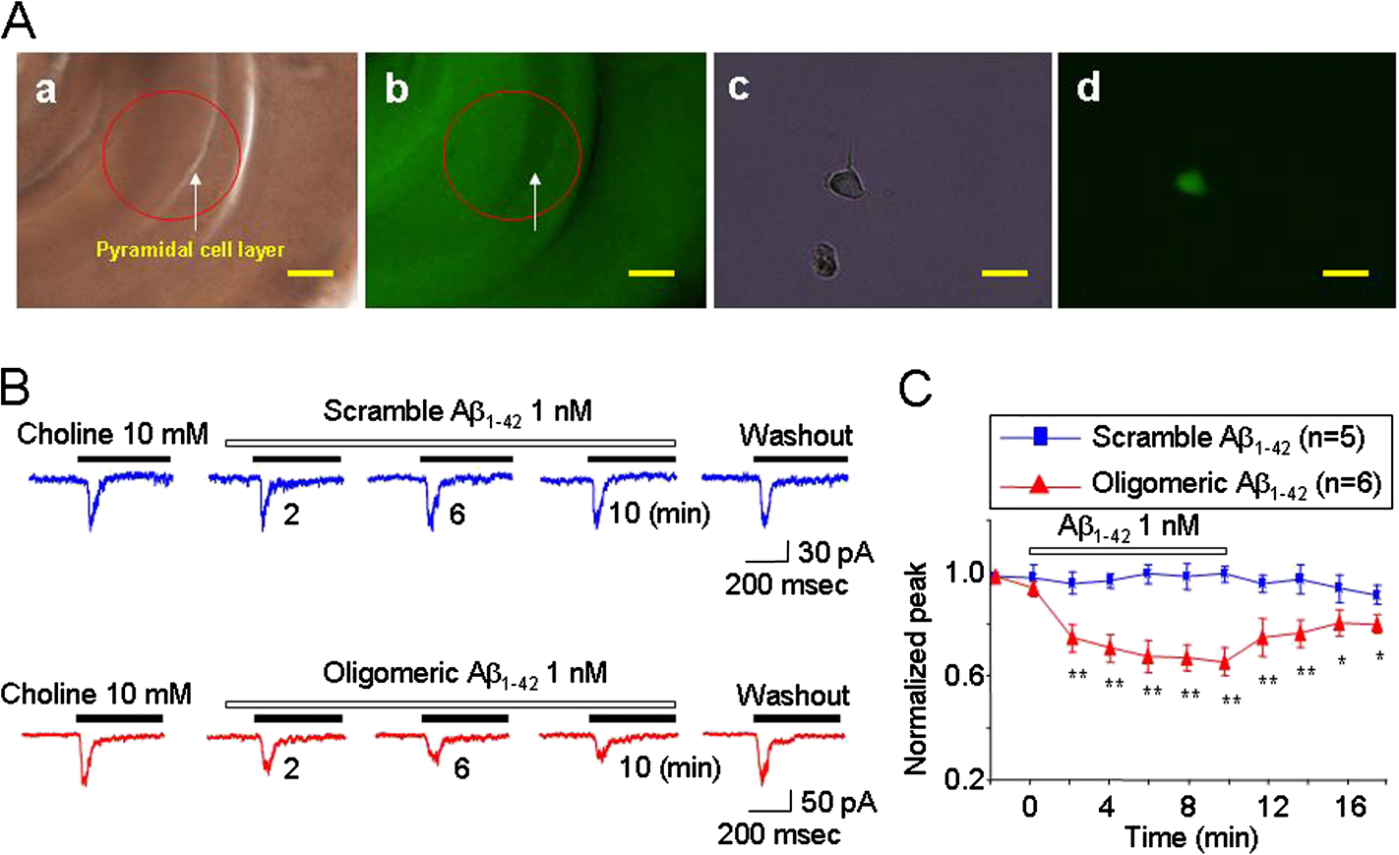
Effects of 1 nM Aβ on α7*-nAChRs in acutely dissociated hippocampal interneurons prepared from GAD67-GFP knock-in mouse. A: Acutely dissociated hippocampal GABAergic interneurons from GAD67-GFP knock-in mice. Phase contrast microscopic image of ventral hippocampus section from a GAD 67-GFP knock-in mouse (**A**a). Red labeled region of ventral hippocampus CA1 indicates the area punched out for acute dissociation. GAD 67-GFP expressing interneurons of ventral hippocampus section from a GAD 67-GFP knock-in mouse display green florescence (Ab). Representative phase contrast (**A**c) and green florescence images of interneuron (Ad) acutely dissociated from the GAD 67-GFP knock-in mouse ventral hippocampal CA1 area. Scale bar = 250 μm in **A**a-b and 10 μm in **A**c-d. **B.** Effects of 1 nM Aβ on choline-induced whole-cell currents in acutely dissociated hippocampal interneurons from GAD67-GFP knock-in mouse. Typical traces illustrate the effects of 1 nM scrambled Aβ (blue traces) or oligomeric Aβ (red traces) on 10 mM choline-induced currents recorded from hippocampal interneurons. The membrane potential was held at −60 mV for these recordings. **C:** Summary of results of experiments as shown in **B**. Each symbol was averaged from 5 or 6 cells tested. The vertical bars indicate Mean ± SE. *p < 0.05, **p < 0.01, *t-test.*

To confirm the effect of Aβ on identified GABAergic interneurons in hippocampus, we examined Aβ’s effect on GFP-expressing cells acutely dissociated from hippocampus of GAD67-GFP knock-in mice (Figure 6Aa-d). As shown in Figure 6Ad, dissociated GABAergic neurons could be easily identified since they exhibited green fluorescence. We then examined and compared the effects of 1 nM scrambled Aβ or 1 nM oligomeric Aβ on 10 mM choline-elicited currents in the identified GABAergic neurons. Choline was repetitively exposed to recorded neuron with an interval of 2 min (Figure 6B). Results from quantitative analysis indicate that choline-induced currents in identified GABAergic neurons are sensitive to 1 nM Aβ exposures (Figure 6C). These data support our findings that putative α7β2-nAChR expressed in hippocampal GABAergic interneurons are sensitive to nanomolar level of oligomeric Aβ.

## Discussion

### Principle findings

The α7-nAChR is traditionally thought as a homomeric receptor [32]. However, we have previously demonstrated the existence of a novel, heteromeric α7β2-nAChR in basal forebrain cholinergic neurons, and the α7β2-nAChRs exhibit high sensitivity to acute Aβ exposure [17]. In this study, we asked whether the heteromeric α7β2-nAChRs are also expressed in hippocampal GABAergic interneurons and whether these receptors are sensitive to pathological levels of Aβ. We found that hippocampal GABAergic interneurons natively express functional α7β2-nAChRs that are highly sensitive to pathologically-relevant concentrations of Aβ. These findings suggest that Aβ could disrupt cholinergic input to hippocampal interneurons to impair neuronal network, which suggests a profound role of these α7β2-nAChRs expressed in hippocampus.

### α7*-nAChRs are predominantly expressed in hippocampal interneurons

There are long-standing disagreements and controversy about the expression profiles of α7-nAChRs in hippocampal neurons. For example, some groups found expression and function of α7-nAChRs in CA1 pyramidal neurons from acutely or organotypically cultured hippocampal slices [33-36], whereas others reported that interneurons, rather than pyramidal neurons, in acute hippocampal slices preferentially express functional nAChRs [37-40]. In the current study, we utilized enzyme dissociation approach to isolate individual neurons from hippocampus CA1 region. After enzyme dissociation, we found that most interneurons express functional α7-nAChRs (79 of 86 neurons tested), however only a small population of pyramidal neurons express functional α7-nAChRs with less discernable choline-induced whole-cell currents (6 of 43 neurons tested). Consistent with above findings [33-40], our data suggest that functional α7-nAChRs are preferentially expressed in CA1 interneurons. Thus, the use of acutely dissociated interneurons to evaluate the alteration of α7-nAChR function after acute Aβ exposure in the present study is appropriate.

### Aβ interacts with α7*-nAChRs in hippocampal interneurons

Aβ accumulation and aggregation in neuritic or senile plaques and severe, selective cholinergic neuronal deficits are two characteristic hallmarks of AD [1]. Many previous findings suggest direct and functionally-relevant interactions of Aβ with α7*-nAChRs [9,13,14,41,42]. Studies of Aβ effects on α7-nAChR function have been seemingly contradictory, perhaps due to differences in experimental protocols used in Aβ studies and variables such as peptide concentrations and forms. Many recent studies show that Aβ directly modulates α7-nAChR function [9,13,17,18,41], and most of these findings, including ours, suggest that acute Aβ exposure directly inhibits α7-nAChR function. Oligomeric Aβ modulates neuronal function more dramatically than monomeric Aβ [43] and has more toxic effects [44]. In the present study, we utilized AFM to monitor the Aβ morphology during its aggregation. Combined with AFM, electrophoresis was used to examine what exact forms of Aβ peptides were present in our samples. We found that after 2 hr aggregation of Aβ, if dissolved with water, the major form was 9-mers in our samples. Nanomolar Aβ concentrations (1–100 nM) are thought to be most pathologically relevant based on levels found in AD patients and in animal models of disease [45]. Aβ oligomers (1 nM equivalent of Aβ monomers) were used in the present study and this concentration is relevant to pathological levels of Aβ in AD brains. The current findings are consistent with our previous observations that functional α7β2-nAChRs are expressed on native neurons and they are sensitive to 1 nM Aβ (equivalent of Aβ monomers) [17], while the similar concentration of Aβ likely does not affect homomeric α7-nAChR function in VTA DA neurons. Together, these results suggest that α7β2-nAChRs are sensitive targets of effects of Aβ exposure.

### Roles of α7-nAChRs in AD pathogenesis and therapy

Significant loss of radioligand binding sites corresponding to nAChRs has been consistently observed at autopsy in a number of neocortical areas and the hippocampus of patients with AD [46,47]. Losses in α7-like-nAChR radioligand binding sites have been reported in several brain regions of AD patients [46]. Decreases in numbers of radioligand binding sites corresponding to α7-nAChRs are among the earliest events detected in AD, preceding cholinergic marker and neuronal loss [46]. Anti-cholinergic signaling is known to impair memory, and nicotine exposure improves cognitive function in AD patients [48], supporting crucial roles for cholinergic signaling and nAChRs in cognitive function. Activation of nAChRs moderates Aβ toxicity, for instance, stimulating nAChRs inhibits amyloid plaque formation in vitro and in vivo [49], activates α-secretase cleavage of amyloid precursor protein (APP) [50], increases ACh release, facilitates Aβ internalization [51], inhibits activity of the MAPK/NF-κB/c-myc pathway [6], reduces A production and attenuates tau phosphorylation [52]. These findings suggest that signaling through nAChRs not only is involved in cognitive function, but also involved in the pathogenic processes in AD.

Hippocampal interneurons have a crucial role in regulating the complex interactions between pyramidal cells and represent a key to the understanding of network operations [53]. Hippocampal interneurons have been reported to highly express α7-nAChRs [28,54], implying an important role played by α7-nAChR in hippocampal function. Aβ and α7-nAChR are both detected in hippocampus in AD patients and amyloid precursor protein (APP) transgenic mice [55-59], accompanied with prevalent loss of hippocampal neurons [60]. Previous findings suggest that acute exposure of hippocampal neurons to high concentrations of Aβ (high nanomolar to low micromolar) inhibits α7-nAChR function [9,13]. In the present study, we found that exposure with physiologically relevant concentrations (e.g., 1 nM) of Aβ oligomers can significantly inhibit α7-nAChR-mediated currents. We think that use of the single neuron preparation and Aβ_1-42_ oligomers may cause this difference of sensitivity. Recent evidence demonstrates that neuronal circuits in hippocampus exhibit hyper-excitation rather than hypo-excitation in both AD patients and APP transgenic animals [21-26]. Palop et al. reported an aberrant neuronal hyper-excitation in APP over-expressing mice models [23,24,27]. It has been reported that the activation of α7-nAChRs expressed on CA1 interneurons enhances inhibitory postsynaptic currents (IPSCs) in the postsynaptic CA1 pyramidal neurons and that these inhibitory responses were blocked by the α7-nAChRs-selective antagonist MLA [29]. Furthermore, the activation of α7-nAChRs expressed on CA1 interneurons produced GABAergic inhibition in nearby pyramidal neurons [33,61,62]. Thus, blockade of α7-nAChRs expressed on CA1 interneurons may lead to disinhibition of pyramidal neurons, while reduced or impaired cholinergic innervations will tune down GABAergic inhibition from GABAergic interneurons to pyramidal neurons [63]. Thus, disruption of cholinergic input to hippocampal GABAergic interneurons might cause disinhibition of pyramidal neurons in hippocampus and then lead to neuronal network hyperexcitation with further deficit in AD.

## Conclusion

Taken together, our findings suggest that functional α7β2-nAChRs are expressed in hippocampal GABAergic interneurons, and these receptors are sensitive to nanomolar concentrations of oligomeric Aβ. The inhibition of α7β2-nAChRs in GABAergic neurons by pathological levels of Aβ may cause acute disruption of cholinergic signaling on interneurons, disinhibition of principal cell types (e.g., pyramidal cells), and ultimately deficits of learning and memory abilities [64]. Moreover, the inhibition of α7β2-nAChR function in interneurons by oligomeric Aβ could also lead to a loss of trophic support for these neurons and accelerate the progression of AD. Drugs targeting α7β2-nAChRs to protect them against Aβ effects or restoration of α7β2-nAChR function may be a new therapeutic strategy for AD treatment.

## Methods

### Animals

Three types of male (PND 14–21) mice (wild-type C57BL/6 mice, nAChR β2 subunit knockout mice on a C57BL/6 background and the glutamate decarboxylase-67 (GAD67)-green fluorescent protein (GFP) knock-in mice on a CD-1 background [65] were used in this study. Experiments were approved by the Institutional Animal Care and Use Committee at the Barrow Neurological Institute, St. Joseph's Hospital and Medical Center. Mice were group-housed in plexiglas shoebox-style cages with ad libitum access to food and water. PCR genotyping was performed to confirm the genetic status of these mice. Genomic DNA from mice newly born to heterozygotic, nAChR β2 subunit knock-out parents was extracted from mouse tail tips by using the QIAgen DNeasy Blood & Tissue Kit following the manufacture's protocol. PCR amplification of the nAChR β2 subunit or lac-Z (an indicator for the knock-out) was performed and PCR products were then resolved on 1% agarose gels and stained for visualization as described previously [17]. Phenotyping of GAD67-GFP knock-in mice was achieved by examining the heads of the mice during postnatal 1–5 days, and these GAD67-GFP knock-in mice exhibited a striking green fluorescence in the brain that can be visualized through the skull at this age, as described previously [66].

### Immunofluorescence staining

Cells were injected with biocytin (5 mg/ml included in the intracellular solution) during patch-clamp recordings for immunostaining in 35 mm culture dishes. After recordings, cells were fixed in 4% paraformaldehyde for 10 min and washed with PBS 3 times at room temperature. Then a PBS-based blocking solution containing 5% normal goat serum and 0.3% Triton X-100 was then applied for 1 hr. After incubations at 4°C overnight with the primary GAD 67 antibody (1:100 dilution; Santa Cruz Biotechnology, Santa Cruz, CA), the cultures were then washed with PBS three times. Thereafter, Avidin (AF488) and GAD 67 secondary antibody (Alexa 555-conjugated, anti-goat) were applied in the blocking solution for 2 hr at room temperature (all used at 1:1000 dilutions; all from Invitrogen, Carlsbad, CA). Cells were then finally washed three times for 5 min with PBS.

### Acutely dissociated neurons from hippocampus and patch-clamp whole-cell current recordings

Neuron dissociation and patch-clamp recordings were performed as described by Wu et al. [17,30,67]. Briefly, postnatal 2 to 4-week-old mice were anesthetized using isoflurane, and the brain was rapidly removed. Several 400 μm coronal slices, which contained the dorsal CA1 region of the hippocampus were cut using a vibratome (Vibratome 1000 plus; Jed Pella Inc., Redding, CA) in cold (2–4°C) artificial cerebrospinal fluid (ACSF) containing (in mM): NaCl, 119; KCl, 2.5; NaHCO_3_, 26; MgSO_2_, 1.3; NaH_2_PO_4_, 1.0; CaCl_2_, 2.5 and glucose, 11, pH = 7.4. The ACSF was continuously bubbled with 95% O_2_ - 5% CO_2_. The slices were then incubated in a chamber (Warner Instruments, Hamden, CT) and allowed to recover for 2 hr at room temperature in oxygenated ACSF. Thereafter, the slices were treated with pronase (1 mg/ml) at 31°C for 30 min and subsequently treated with protease (1 mg/ml) for another 30 min. The ventral CA1 region was extracted by punching slices using a well-polished needle. The punched tissue was then dissociated mechanically by using several fire-polished micro-Pasteur pipettes in a 35 mm culture dish filled with oxygenated standard external solution [in mm: 150 NaCl, 5 KCl, 1 MgCl_2_, 2 CaCl_2_, 10 glucose, and 10 HEPES; pH 7.4 (with Tris-base)]. Perforated-patch whole-cell recordings coupled with a three-barrel drug application system were used (Warner Instruments, Hamden, CT). To prepare for perforated-patch whole-cell recording, glass microelectrodes (GC-1.5; Narishige) were fashioned on a two-stage vertical pipette puller (P-830; Narishige, NY, USA), and the resistance of the electrode was 4–6 MΩ when filled with the internal solution. A tight seal (>2 GΩ) was formed between the electrode tip and the cell surface, which was followed by a transition from on-cell to whole-cell recording mode due to the partitioning of amphotericin B (200 μg/ml, Sigma, St. Louis, MO) into the membrane underlying the patch. After whole-cell, an access resistance lower than 60 MΩ was acceptable for perforated-patch recordings under voltage-clamp mode. The series resistance was not compensated in the experiments using dissociated neurons. Data were acquired by Axopatch 200B amplifier at 5 kHz with pClamp 9.2 software (Molecular Devices, Sunnyvale, CA) and analyzed with Clampfit 9.2 software (Molecular Devices, Sunnyvale, CA).

### Drugs and Aβ preparation

Drugs used in this study were choline, methyllycaconitine (MLA), dihydro-β-erythroidine (DHβE) (Sigma, St. Louis, MO), brefeldin A (Calbiochem, San Diego, CA), scramble Aβ_1-42_, and Aβ_1-42_ (rPeptide, Athens, GA). Aβ_1–42_ was reconstituted in distilled water to a concentration of 100 μM and stored at −80°C as previously described [17]. Aβ was used within 7 days after reconstitution. Aliquots diluted in standard extracellular solution yielded a predominantly oligomeric form. AFM was used to monitor aggregation forms of Aβ. For each use, Aβ stock (100 μM) was then diluted into desired concentrations. In this study, 1 nM Aβ within 4 hr after preparation mostly forms smaller oligomers. In all experiments within this study, Aβ was used within 4 hr before discarded each time.

### Atomic force microscope (AFM) imaging

AFM was used to monitor the morphology of the Aβ aggregates before experiments. Aliquots were removed from Aβ samples, and then immediately spotted on freshly cleaved mica. After 2 min the mica was washed with 1 ml of de-ionized water, and then dried with compressed nitrogen. Topographic AFM images were obtained in air at room temperature using a Tapping Mode AFM with a Nanoscope IIIa controller (Veeco, Santa Barbara, CA). Images were acquired using oxide sharpened Si_3_N_4_ AFM tips (k = 40 N/m, f_o_ ~ 300kHz) (Model: OTESPA, Veeco, Santa Barbara, CA) at scan rates of 2–3 Hz and at scan resolution of 512 samples per line. Images were subjected to 2^nd^ order polynomial flattening as needed to reduce the effects of image bowing and tilt. AFM images were analyzed with the Scanning Probe Imaging Processor (SPIP) software (Image Metrology, http://www.imagemet.com) to generate height distribution histograms for each sample.

### Immunoprecipitation and electrophoresis

Tissues were Dounce homogenized (10 strokes) in ice-cold lysis buffer [1% (v/v) Triton X-100, 150 mm EDTA, 10% (v/v) glycerol, 50 mm Tris–HCl, pH 8.0] containing 1× general protease inhibitor cocktails (Sigma-Aldrich, St. Louis, MO). The lysates were transferred to microcentrifuge tubes and further solubilized for 30 min at 4°C. The detergent extracts (supernatants) were collected by centrifugation at 15,000 × *g* for 15 min at 4°C, and protein concentration was determined for sample aliquots using bicinchoninic acid (BCA) protein assay reagents (Pierce Chemical, Rockford, IL). The detergent extracts were then precleared with 50 μl of mixed slurry of protein A-Sepharose and protein G-Sepharose (1:1) (Amersham Biosciences, NJ) twice, each for 30 min at 4°C. Detergent extracts were mixed with 1 μg of rabbit anti-α7 antiserum (H302, Santa Cruz Biotechnology, Santa Cruz, CA) and incubated at 4°C overnight with continuous agitation. Protein A-Sepharose and protein G-Sepharose mixtures (50 μl) were added and incubated at 4°C for 1 hr. The beads were washed four times with ice-cold lysis buffer containing protease inhibitors. Laemmli sample buffer eluates were resolved by SDS-PAGE. Proteins were transferred onto Hybond ECL nitrocellular membranes (Amersham Biosciences, NJ). The membranes were blocked with TBST buffer [20 mm Tris–HCl, pH 7.6, 150 mm NaCl, and 0.1% (v/v) Tween 20 containing 2% (w/v) nonfat dry milk for at least 2 hr and incubated with rat monoclonal anti-β2 antibody (mAb270; Santa Cruz) or rabbit anti-α7 antiserum (H302), respectively, at 4°C overnight. After three washes in TBST, the membranes were incubated with goat anti-rat or goat anti-rabbit secondary antibodies (1:10,000) (Pierce Chemical, Rockford, IL) for 1 hr and washed. The bound antibodies were detected with SuperSignal chemiluminescent substrate (Pierce Chemical, Rockford, IL).

Aβ _1–42_ peptides were analyzed with electrophoresis to test the exact form of oligomer during aggregation state. Pre-cast 10-20% SDS-polyacrylamide Tris-Tricine gels (Bio-Rad, Hercules, CA) or 16% Tris-Tricine gels in the presence or absence of SDS or Urea 8M were used. 100 μg of Aβ_1-42_ per sample was resuspended with 4X Tricine loading buffer. Aβ_1-42_ samples dissolved with water or DMSO were aggregated for 2 hr before loaded.

### Statistical analysis

All data were presented as mean ± standard error (SE). Statistical comparisons using Student’s t-test (independent or paired) were performed with Origin 5.0 (Microcal Software, Inc., Northampton, MA). *p* values less than 0.05 were considered statistically significant.

## Abbreviations

nAChRs: nicotinic acetylcholine receptors; Aβ: amyloid β peptide; DHβE: dihydro-β-erythroidine; AD: Alzheimer’s disease.

## Competing interests

The authors declare that they have no competing interests.

## Authors’ contribution

QL: Performed experiments, analyzed data, and wrote manuscript. YH: Performed experiments using immunoprecipitation, and revised manuscript. JXS: Designed experiments and revised manuscript. SS: Designed experiments and revised manuscript. JW: Designed experiments, performed some patch-clamp recordings and wrote manuscript. All authors read and approved the final manuscript.
